# Road Extraction from High Resolution Remote Sensing Images Based on Vector Field Learning

**DOI:** 10.3390/s21093152

**Published:** 2021-05-01

**Authors:** Peng Liang, Wenzhong Shi, Yixing Ding, Zhiqiang Liu, Haolv Shang

**Affiliations:** 1 School of Remote Sensing and Information Engineering, Wuhan University, Wuhan 430072, China; liangpeng1984@whu.edu.cn; 2 Department of Land Surveying and Geo-Informatics, The Hong Kong Polytechnic University, Hong Kong, China; 3 Key Laboratory of Digital Earth Science, Aerospace Information Research Institute, Chinese Academy of Sciences, Beijing 100094, China; dingyx@radi.ac.cn (Y.D.); shanghl@radi.ac.cn (H.S.); 4 Piesat Information Technology Co., Ltd., Beijing 100195, China; lzq754802201@163.com

**Keywords:** road extraction, vector field learning, high resolution remote sensing image, encoder-decoder, DCNN

## Abstract

Accurate and up-to-date road network information is very important for the Geographic Information System (GIS) database, traffic management and planning, automatic vehicle navigation, emergency response and urban pollution sources investigation. In this paper, we use vector field learning to extract roads from high resolution remote sensing imaging. This method is usually used for skeleton extraction in nature image, but seldom used in road extraction. In order to improve the accuracy of road extraction, three vector fields are constructed and combined respectively with the normal road mask learning by a two-task network. The results show that all the vector fields are able to significantly improve the accuracy of road extraction, no matter the field is constructed in the road area or completely outside the road. The highest F1 score is 0.7618, increased by 0.053 compared with using only mask learning.

## 1. Introduction

Roads are important transportation facilities. Accurate and up-to-date road network information is of great significance for the Geographic Information System (GIS) database, traffic management and planning, automatic vehicle navigation, emergency response and other applications [1]. Road networks can also represent the spatial distribution of automobile exhaust, a major urban air pollutant. Therefore, real-time acquisition technologies for up-to-date road information are urgently needed. Meanwhile, with the rapid development of remote sensing technology, massive high-resolution images are accessible to the public and provide sufficient and high-quality data for road automatic extraction. Artificial intelligence gives us access to powerful tools with fast speed and high accuracy. Thus, road extraction from high resolution remotes sensing images has become one of the research hotspots in the fields of photogrammetry, remote sensing, computer vision and geographic information science.

At present, there are many methods of extracting road from remote sensing imaging, which can be roughly divided into several categories: tracking methods, morphology and filtering methods, methods based on dynamic programming and active contour model, multi-scale analysis methods, knowledge expression and fuzzy-model-based methods, pixel segmentation methods, region segmentation methods, edge detection methods, Markov random field (MRF) and conditional random field (CRF) methods, traditional classification methods and Deep Convolution Neural Network (DCNN) methods. Among those methods, the DCNN is characterized by the automatic learning of the deep hierarchical representation from data. In, different fields such as scene recognition, target detection and semantic segmentation, the DCNN shows better performance and greater potentiality compared with traditional methods. The full convolutional network (FCN), U-net and Generative Adversarial Networks (GAN) are DCNNs widely used in road extraction. FCN, which is a “fully convolutional” network that uses the interpolation layer after the final convolution layer for up-sampling. It takes input image of arbitrary size and produces correspondingly sized output with efficient inference and learning [2]. For the literature concerning road extraction using FCN, refer to [3,4,5]. U-net is a popular U-shaped encoder-decoder network, which shows excellent performance in medical and remote sensing image segmentation [6]. An important modification of FCN by U-net is that there are a large number of feature channels in the up-sampling part, which allows the network to propagate context information to higher resolution layers. The research ideas of road extraction using U-net include multivariate loss function [7,8], modification, of network architecture such as new network unit or adding jump connection [9,10,11,12,13], pre-training [14], multitask learning strategy [15,16], etc. In addition to U-net, some new encoder-decoder networks are also proposed for road extraction [17,18,19,20]. Luc et al. [21] introduced GAN into the field of semantic segmentation. Its basic idea is to train a convolutional semantic segmentation network (generator) and an adversarial network (discriminator). Costea et al. [22] proposed a two-stage method to identify road intersections. Varia et al. [23] used a conditional GAN model to extract roads from images. Zhang et al. [24] proposed a road extraction network based on Multi-supervised Generative Adversarial Network (MsGAN), which is trained by the spectrum and topological characteristics of the road network. In addition, the appropriate pre-processing or post-processing technology is sometimes more effective than modifying the network in improving the accuracy. For related research, please refer to [25,26,27]. 

The idea of vector field learning comes from the deep watershed transform [28], which has been applied in some related visual tasks. There are also some other related studies. DeepFlux [29] is a method of extracting natural target skeleton. It constructs a vector field in the region of interest. The vector of pixel in the region of interest points to the nearest skeleton pixel, and the vector of skeleton and background pixel is 0. Textfield [30] is used for text segmentation, and the vector of pixel in the region of interest points to the nearest edge pixel. Super BPD [31] is similar, but the region of interest extends to the entire image. Batra et al. [32] propose a connectivity learning task which uses the road segment orientation vector. These studies prove that the vector field is powerful in describing the shape and overall structure of an object in a complex background.

Basically, there are three ways of learning the road area: using the semantic segmentation method to learn the mask, learning the centerline and width of the roads and extracting the edge of the road area. Here we will give the fourth choice: learning the vector field of the road. In this paper, the idea of vector field learning, to our knowledge, is first introduced in road extraction. Specifically, the main contribution of this paper is that we construct three different learnable vector fields to describe the road centerline, road area and road background respectively. The first vector field uses the concept of road segment orientation, but we give all pixels in one segment the same orientation. The second vector field is similar to DeepFlux, which is constructed in the road, area except for the centerline. The centerline is considered the skeleton of the road. The third field is simply constructed in background areas. A two-task network is used to combine mask learning and vector field learning. The definition of three vector fields, as well as the network structure, will be present in Section 2. The results, shown and discussed in Section 3, will prove that all three vector fields can help improve the F1 score.

## 2. Materials and Methods

### 2.1. Data Source

**Figure 1:**
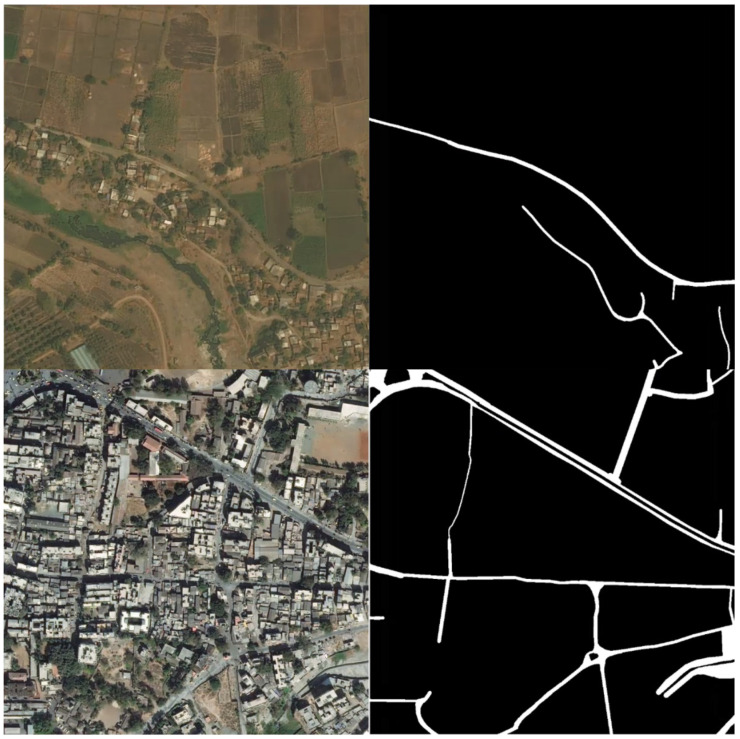
The example images and their labels.

The dataset used in this paper is DeepGlobe 2018 [33], which contains 6226 training images, 1243 validation images and 1101 test images and spans a total area of 2220 km^2^. All images are 1024 × 1024 in size and 0.5 m in spatial resolution, and consist of 3 channels (Red, Green and Blue). The images are from India, Indonesia and Thailand. The image scenes include urban, suburban, rural, tropical rain forest, seashore and other landforms. This dataset is sampled from the larger dataset, DigitalGlobe Basemap +Vivid. The sources of data include WorldView-2, Worldview-3 and Geoeye-1. The average image age is targeted to be smaller than 30 months. Since the label of validation images and test images are inaccessible, we divided the training data into two parts, 4000 images for training and the remaining 2226 images for testing. Figure 1 shows two examples, one depicting rural areas and the other describing urban areas. It can be seen from the images that the surface material, geometrical shape and denseness of road vary greatly in different images. Besides that, the images also demonstrate sheltered roads by trees and buildings, and other, road-like linear targets such as the bank of a river, the ridge of a field and the edge of a building. Therefore, this dataset is suitable for testing our methods in this case. The dataset can be downloaded at the website https://competitions.codalab.org/competitions/18467#participate (Accessed on 10 March 2021). 

### 2.2. Methods

**Figure 2:**
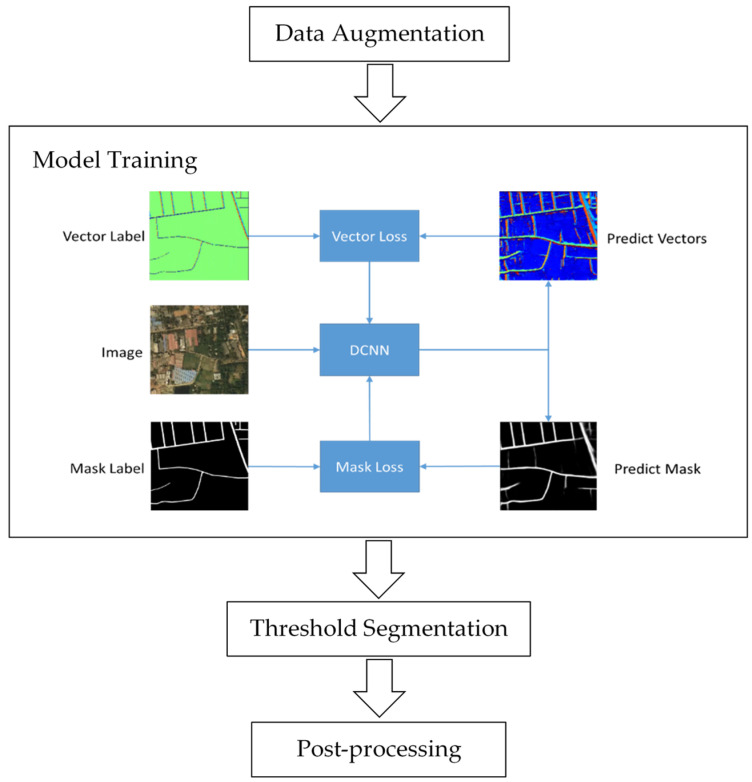
The simplified flow chart of our experiment. Three kinds of vector fields are constructed and compared.

In this section, the field learning for road extraction will be introduced in detail. The overall flowchart is simplified in Figure 2. At first, we use common data augmentation methods to expand the training dataset. Then the network is trained by the mask and vector fields, which will be introduced in 3.2.2. In our experiment, three kinds of vector fields are constructed and tested. After network output, the threshold segmentation and post-processing are followed to improve the results.

#### 2.2.1. Data Augmentation

**Figure 3:**
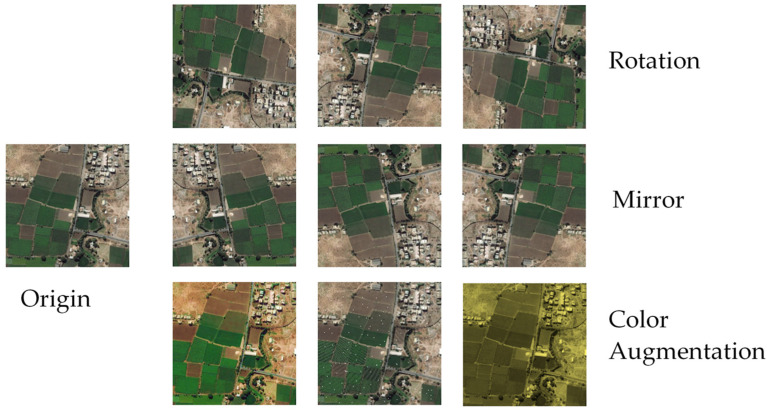
Data Augmentation. Three data augmentation methods are used. On the right side of the figure are 9 augmented images. In the first line, the three images are rotated 90°, 180° and 270°, respectively. In the second line, the three images are horizontally mirrored, vertically mirrored and centrally mirrored, respectively. In the third line, the three images are saturation enhanced, randomly noised and color adjusted, respectively.

Training DCNN needs a lot of samples. Generally, the more sample data used, the better performance the model has. However, in practice, the number of samples is limited by the dataset. Therefore, in order to make the best use of existing data resources, we expand the training samples by data augmentation. The used data augmentation methods are (Figure 3):

1. rotation, rotating the image randomly 90°, 180°, 270°;
2. mirror, including horizontal mirror and vertical mirror;
3. random color augmentation, such as changing the image pixel value, adjusting brightness, saturation and so on.

#### 2.2.2. Vectors Field for Road Extraction

The vector field are widely used in describing the dynamics or distribution of nature objects. The wind field in the atmosphere and the current in the ocean are expressed by direction and intensity, which can be used to divide different regional climate characteristics and ocean current models. The orientation field contains information crucial to the fingerprint recognition. Furthermore, in skeleton extraction from natural images, vector fields are powerful tools. Unlike the gradient field, these vector fields are always directly related to the shape and structure of the object. In computer vision, an object can be represented by skeleton information. Skeleton is composed of a series of points which contains the shape characteristics. There are some similarities between image skeleton extraction and road extraction. Skeleton and road are both continuous and directional linear structures, and each part has a topological relationship. The centerline can be regarded as the skeleton of the road. To better present the construction of three vector fields, we first divide all the pixels in the image into three categories: road centerline pixels, non-centerline road pixels (road pixels but not on the centerline) and background pixels. We use C, R and B to represent the set of pixels in the three categories respectively. It can be learned from the experience of skeleton learning that there are close relationships among three kinds of pixel, and we use these relationships to construct three vector fields. In this paper, to construct a vector field for an image means giving each pixel in this image a corresponding vector, and the vector will be recorded in a two-layer image which has the same size as the original image. 

(1) Centerline Vector Field (CVF)

**Figure 4:**
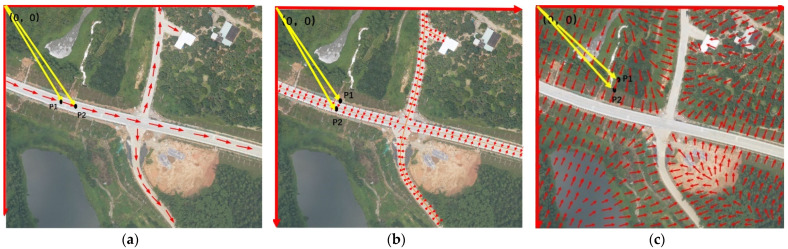
Three Vector Fields for Road Extraction: (**a**) CVF, (**b**) RVF and (**c**) BVF. The long red arrows indicate the axes of image coordinate systems, the long yellow arrows indicate the image coordinates of any pixels in the image, and the short red arrows indicate the unit vectors in the fields.

CVF is a directional field constructed along the road extension direction. According to the label of road samples, we use morphological method to obtain the label of road centerline, and then calculate its nodes and intersections. The nodes and intersections can divide the net of the centerline into road lines. Because the points on the road lines have different directions, we further divide the road lines into shorter line segments with a certain length threshold. If the threshold is short enough, the direction of each line segment d can be defined by the vector that points from the starting pixel to the ending pixel of the line segment. All pixels on a line segment are given the same vector. After all centerline pixels are given corresponding vectors, the CVF in Figure 4a can be obtained. In practice, the road has a certain width. Therefore, we expand the CVF from the road centerline to the whole road area by assigning the non-centerline road pixel with the vector of centerline d. Then, the vector field $f_{c}$ can be given by:

(1) $$f_{c}\left(p\right)=\{\;\;\begin{array}{cc} d, & p\in C\cup R\\ \left(0,0\right) & \;p\in B \end{array}$$

(2) Road Vector Field (RVF)

RVF is a vector field constructed for the non-centerline road pixels. All vectors of background pixels and road center pixels are set to (0,0), while the vector of each non-centerline road pixel is pointing to its nearest road center pixel (Figure 4b). The vector field $f_{r}$ can be given by:

(2) $$f_{r}\left(p\right)=\{\;\;\begin{array}{cc} p_{c}-p, & p\in R,p_{c}\in C\\ \left(0,0\right) & p\in C\cup B \end{array}$$

where $p_{c}$ is the nearest pixel of p in C.

(3) Background Vector Field (BVF)

BVF is a vector field constructed for road background. In the image, the vector of each pixel in road area is set to (0,0), and the vector of each background pixel is pointing to its nearest road pixel, namely the nearest zero pixel. By calculating the vectors of all background pixels in turn, the BVF in Figure 4c can be obtained. The vector field $f_{b}$ can be given by:

(3) $$f_{b}\left(p\right)=\{\;\;\begin{array}{cc} p_{r}-p, & p\in B,\;p_{r}\in R\;\\ \left(0,0\right) & p\in C\cup R\; \end{array}$$

where $p_{r}$ is the nearest pixel of p in R.

#### 2.2.3. Network

**Figure 5:**
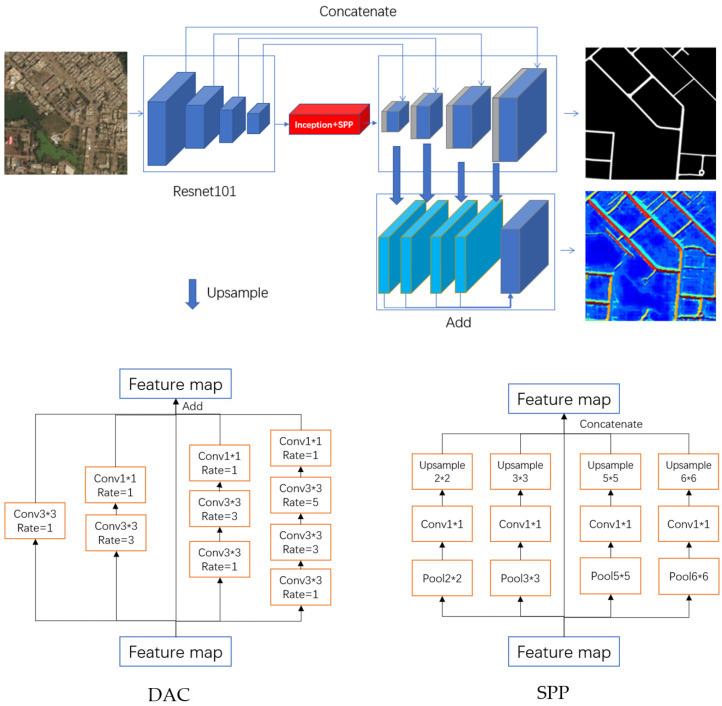
The Structure of the Network for Road Extraction.

The network used in this paper is an encoding-decoding structure. Resnet101 is used as the backbone in the encoding process. The dense atrous convolution (DAC) and spatial pyramid pooling (SPP) modules are added deeply in the network to fully excavate the deep features of the road [34]. Then, the data are up-sampled by deconvolution, and fused with outputs of the middle layers in the decoding process. The fusion results are output in two forms. One is the road mask map, and the other is the road vector field map. It is a two-task learning strategy (Figure 5). 

(1) Resnet

The network input size is 1024 × 1024. Firstly, the input image is processed by a 7 × 7 convolution with a step size of 2 to output a 512 × 512 feature layer. Then, after processed by the first residual block, the 256 × 256 feature layer is obtained. Similarly, after several residual blocks, feature layers with the sizes of 128 × 128, 64 × 64, 32 × 32 and 16 × 16 are obtained successively. Resnet is chosen for its relative ease of optimization and ability to improve the accuracy from the increased depth.

(2) DAC

This module has five cascade branches which employs different receptive fields. The convolution of different reception fields is used to extract more abstract features for roads with different width. The large reception fields are better for wide roads, while the small reception field are better for narrow roads.

(3) SPP

This module is also composed of five branches. The input feature map is pooled and up-sampled in five different scale branches (1, 2, 3, 5 and 6), and concatenated together to get the final feature map. The calculation cost of this module is very low. Through pooling of different scales, the same features are merged, and the different features are separated, which is helpful to merge and eliminate the background information.

#### 2.2.4. Training

Generally speaking, the pixels close to the road contain more information about the road, but the pixels far away from the road have a larger vector modulus. In order to balance the weight of different vectors, we need to normalize the vector field before training. 

As initialization, we chose the Resnet which has been pre-trained on Imagenet. Due to the limited computing resources, the extracted middle layer channels are compressed to 64, 128, 256 and 512.

The network has two outputs, corresponding to two tasks, and each task has its own loss function. The weighted binary cross entropy is chosen for road mask learning:

(4) $$L_{m}=-\frac{1}{N}\overset{N}{\underset{i=1}{\sum }}[y_{i}\log p_{i}+\left(1-y_{i}\right)\log\left(1-p_{i}\right)]$$

where N is the number of pixels in the input image, $y_{i}$ is the true value in mask label and $p_{i}$ is the predicted value output by the network.

The sum of weighted 2-norm is chosen for vector learning:

(5) $$L_{v}=\sum w\left(p\right)*\|f\left(p\right)-\hat{f}{\left(p)\right\|}_{2}$$

where w(p) is the weight, f(p) is the true vector in the vector label and $\hat{f}\left(p\right)$ is the predicted vector output by the network. The w(p) is defined by:

(6) $$w\left(p\right)=\{\;\;\begin{array}{cc} \frac{\left|B\right|}{\left|R\right|+\left|B\right|+\left|C\right|}, & p\in R\cup C\;\\ \frac{\left|R\right|+\left|C\right|}{\left|R\right|+\left|B\right|+\left|C\right|}, & p\in B \end{array}$$

The weight w(p) is introduced to reduce the impact of the imbalance between the number of road pixels and background pixels.

In the two-task framework, the total loss function is given by:

(7) $$L=w_{m}L_{m}+w_{v}L_{v}$$

where $w_{m}$ and $w_{v}$ are weight of mask loss function and vector loss function respectively. They are both set to 1 in our experiments.

#### 2.2.5. Post-Processing

(1) Corrosion-expansion and connected component filtering

The common post-processing methods include corrosion-expansion, open-close operation and removing the irregular edges in the image. Connected component filtering is to calculate the adjacency relationship between adjacent pixels in the binary graph. In this paper, we use the 8-neighborhood algorithm to delete some separated points or areas to improve the accuracy.

(2) Regularization

Generally speaking, the road is a continuous and smooth strip. The road extracted from the image will inevitably lead to irregular boundaries due to occlusion or background interference. Here, a road smoothing method is used to regularize the road. When the binary road is obtained, the road centerline is extracted by morphological method, and the width of each point on the centerline is calculated and recorded. At the same time, the road is divided into several segments by a certain length threshold, and the width of each segment of the road is smoothed to get a reliable width data. Finally, the regularized road is recovered by the width data and centerline.

## 3. Results and Discussion

In this section, the results of the four experiments are presented. We first use the network to learn the road mask directly as a reference experiment. Then, the CVF, RVF and BVF are added into our two-task learning framework respectively. For better evaluation, the images presented in this section are not segmented by threshold or post-processed. Thus, the blur still exists in the output image. The evaluating indicator in the tables are calculated after threshold segmentation.

All experiments run on a workstation with a single RTX 3090 GPU, 128GB RAM, and an Intel Xeon 6226r CPU. We use the platform Pytorch and the network optimizer Adam. The initial learning rate is 1e-3, and the weight decay is 1e-4. In the training process, a batch of sample data is input in each step. Every 100 steps, the loss of training data is printed, and every 1000 steps, the loss of verification data is printed. The learning rate is adjusted in the training process, and when the loss of verification data does not drop for several times, the training stops.

### 3.1. Road Extraction Using Mask Learning Only

**Figure 6:**
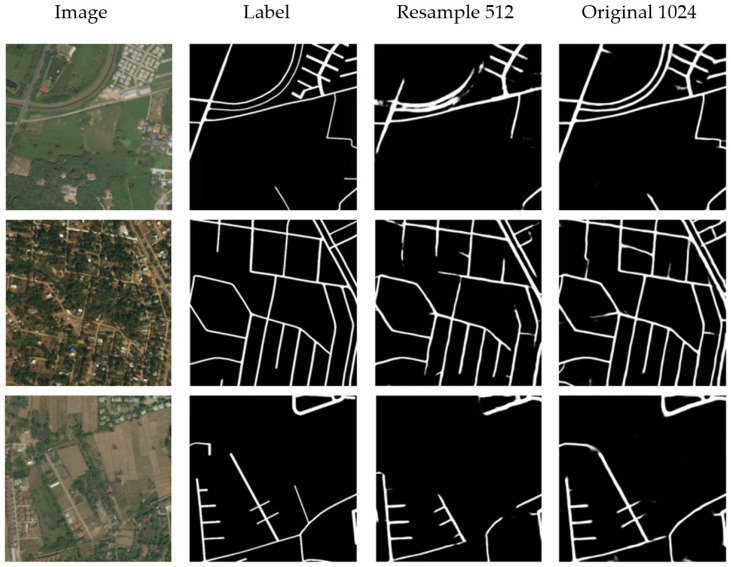
Road Extraction Results Using Mask Learning Only.

The results of direct mask learning are shown in Figure 6 and Table 1. The 1024 × 1024 images are resampled to 512 × 512, and thus two kinds of image resolution can be tested. It is obvious that the extraction result of high-resolution image is much better than that of low-resolution image. In the output with lower resolution, the cut-off and blurring are easier to occur. For example, considering that the original resolution of the dataset is 0.5 m, a 5 m-wide road will be compressed to only 5 pixels in the resampled images. After the multi-layer convolution and pooling operation by Resnet, some features of narrow road are likely to be lost and the useful features cannot be accurately captured. Therefore, the precision, recall and F1 score of high-resolution output are all better than those of low-resolution output. As a reference of following experiment, the F1 score of the mask learning with 1024 × 1024 input is 0.7089.

### 3.2. Road Extraction Using Mask and CVF Learning

**Figure 7:**
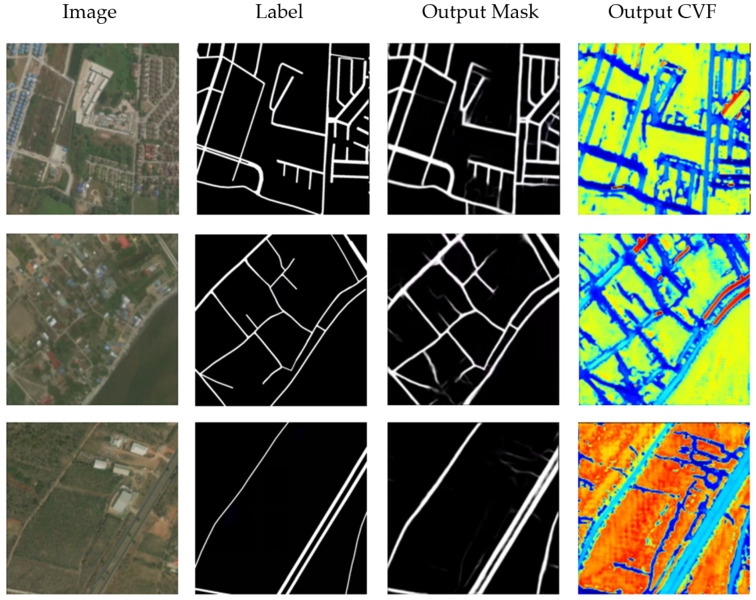
Road Extraction Results Using Mask and CVF Learning.

The vector of any pixel in the road contains the structure information of the road and the gray variation information of the nearby area, so it can help to express the road features more completely. The two-task network can simply combine the loss functions with weighted summation, which reduces the risk of over-fitting. At the same time, the mask can be directly output, rather than recovering from the vector field by extra algorithm. Figure 7 and Table 2 show the results of mask and CVF learning. The network outputs two results, namely the road map and the vector field map. After adding vector field learning, the F1 score is improved to 0.7575, which indicates that direction features of road can greatly improve the accuracy of road extraction. It can also be seen from the figure that the roads are more coherent, and the occurrence of cut-off is reduced. However, there are still some problems. The edge of the road, especially at the cross-section and turning point, is not well learned. That may partly attribute to inflection point introduced by road division (see 2.2.2). In some areas, the roads are falsely extended.

### 3.3. Road Extraction Using Mask and RVF Learning

**Figure 8:**
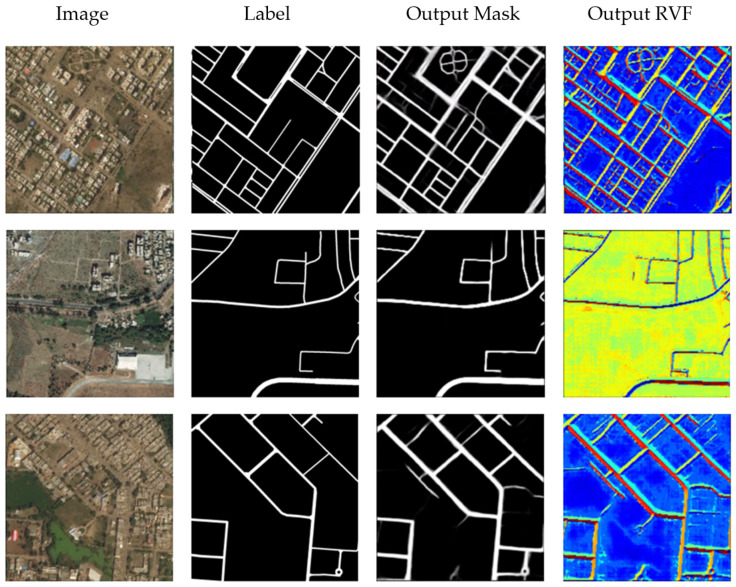
Road Extraction Results Using Mask and RVF Learning.

Figure 8 and Table 3 show the results of mask and RVF learning. It can be seen from the output RVF image that the road edge is well learned compared with the output of CVF, but the false extension is still a problem. It is caused by the similar linear objectives (the rivers, for example) and will be eliminated to some extent after threshold segmentation and post-processing. The F1 score of the mask and RVF learning is 0.7618, slightly better than that of mask and CVF learning. This result may also indicate that the pixel value variation across the road contains more information than that along the road for road extraction. 

### 3.4. Road Extraction Using Mask and BVF Learning

**Figure 9:**
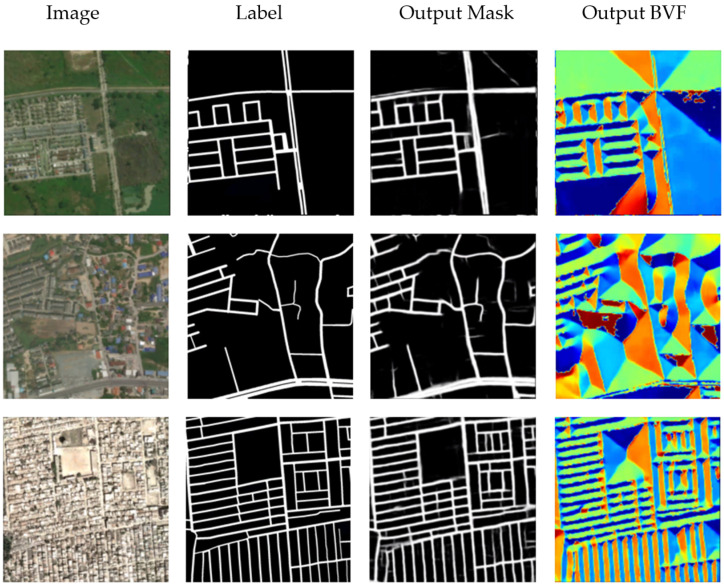
Road Extraction Results Using Mask and BVF Learning.

The previous experiments show that learning the vector field constructed in the road area is very effective to improve the accuracy of road extraction. Then, a new problem arises whether the road external vector field is helpful to improve the accuracy of road extraction. BVF is a vector field completely built outside the road area. After vector normalization, the contribution to the loss function of pixels close to the road will be much greater than that of pixels far away from the road. The corresponding results are shown in Figure 9 and Table 4, which shows that BVF is also helpful to improve the accuracy of road extraction. The F1 score was 0.7588.

### 3.5. Further Discussion

**Figure 10:**
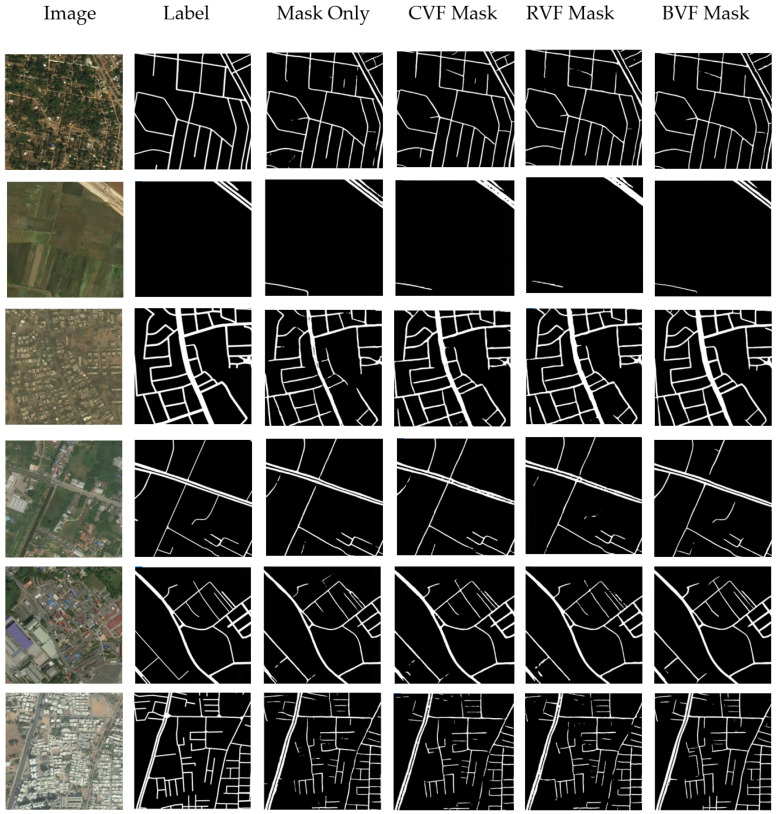
The comparison of different methods. The threshold segmentation of image is carried out.

The results of four experiments are given in the previous section. It can be seen from the indicators in Table 1, Table 2 and Table 3 that vector field learning is beneficial to improve the accuracy of road extraction. Both the vector field inside the road and the vector field outside the road contain a lot of road related information. Generally, the F1 score of RVF learning, BVF learning and CVF learning are very close. In addition, from Table 1, Table 2 and Table 3 and Figure 10, we can find that the results of CVF and RVF are more similar, while the results of BVF are relatively different. This is because CVF and RVF use more road internal information, while BVF use more road external information. The recalls of CVF and RVF are close, but they are significantly higher than that of BVF, and their precisions (also very close) are significantly lower than that of BVF. This may show that road extraction with external information is more robust to the interference of similar targets.

The main purpose of this work is giving a new way to describe the road in high resolution remote sensing images, so we only use a mask learning method as reference. Admittedly, the method in this paper is not the best performed one for road extraction. It is not the original intention of this paper to deal with each step delicately for the best accuracy. We also test other methods. Among them, the D-linknet [35] has the best performance, which gets an F1 score of 0.7696 on the same dataset, slightly better than the result of mask and RVF learning. However, we think that it will not weaken our conclusion and that the vector field learning is still a promising method that is worthy of further study.

**Figure 11:**
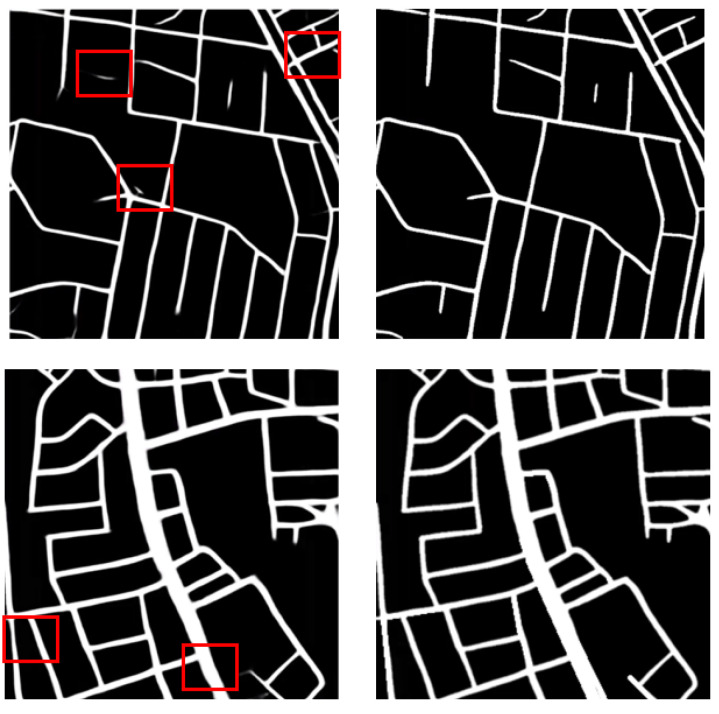
Road Extraction Results Before and After Post-processing.

The purpose of post-processing is to remove some obvious errors through morphological operations and topological relations. The first line of Figure 11 shows some examples of handling topology errors, mainly the removal of some discrete links. The second line of Figure 11 shows some examples of morphological operations to smooth road edges to make the road width more consistent. The post-processing operation can not only make the road network map smoother and closer to the label visually, but also slightly improve the extraction accuracy. After the post-processing operation, the F1 score can be increased by 0.005–0.01.

## 4. Conclusions

In this paper, road extraction from high-resolution remote sensing image is realized by vector field learning. In order to improve the accuracy of road extraction, three vector fields, CVF, RVF and BVF, are constructed, and road extraction experiments are carried out by using a two-task network. The results show that the three vector fields constructed in this paper can significantly improve the accuracy of road extraction. Among them, road extraction accuracy of RVF is the highest, and its F1 score is 0.7618. Furthermore, different from CVF and RVF, BVF is a vector field completely built outside the road, which can also greatly improve the accuracy of road extraction. Our result shows that the texture information inside the road and the texture information outside the road are both very important for road extraction. Moreover, we also find that BVF is more resistant to the interference of road-like targets than CVF and RVF. The later work can focus on how to combine the road internal field and external field to improve the extraction accuracy.

## Figures and Tables

**Table 1:** The performance evaluation of road extraction using mask learning only.

Method	Precision	Recall	F1 Score
Mask Learning_512	0.6942	0.7076	0.6704
Mask Learning _1024	0.7584	0.7332	0.7089

**Table 2:** The performance evaluation of road extraction using mask learning and CVF.

Method	Precision	Recall	F1 Score
Mask and CVF Learning	0.7314	0.8201	0.7575

**Table 3:** The performance evaluation of road extraction using mask learning and RVF.

Method	Precision	Recall	F1 Score
Mask and RVF Learning	0.7315	0.8258	0.7618

**Table 4:** The performance evaluation of road extraction using mask learning and BVF.

Method	Precision	Recall	F1 Score
Mask and BVF Learning	0.7437	0.8060	0.7588

## Data Availability

Not applicable.
